# Supplementary material for: Quasi-continuous production of highly hyperpolarized carbon-13 contrast agents every 15 seconds within an MRI system
Source: Commun Chem. 2022 Feb 18;5:21. doi: 10.1038/s42004-022-00634-2 (PMC9814607; doi:10.1038/s42004-022-00634-2)
Supplement: Supplementary file 1 — Supplementary Information [file 42004_2022_634_MOESM1_ESM.pdf]

Supplementary Information

Quasi-continuous production of highly hyperpolarized carbon-13 contrast agents every 15 seconds within an MRI system

Andreas B. Schmidt[1,2,3,*], Mirko Zimmermann[1], Stephan Berner[1,2], Henri de Maissin[1,2], Christoph A. Müller[1,2], Vladislav Ivantaev[1], Jürgen Hennig[1], Dominik v. Elverfeldt[1], Jan-Bernd Hövener[3,*]

1 Department of Radiology, Medical Physics, Medical Center, University of Freiburg, Faculty of Medicine, University of Freiburg, Killianstr. 5a, Freiburg 79106, Germany.

2 German Cancer Consortium (DKTK), partner site Freiburg and German Cancer Research Center (DKFZ), Im Neuenheimer Feld 280, Heidelberg 69120, Germany.

3 Section Biomedical Imaging, Molecular Imaging North Competence Center (MOIN CC), Department of Radiology and Neuroradiology, University Medical Center Kiel, Kiel University, Am Botanischen Garten 14, 24118, Kiel, Germany.

E-Mail: andreas.schmidt@uniklinik-freiburg.de ; jan.hoevener@rad.uni-kiel.de

Table of Contents

Supplementary Methods 3

A. Preparation of samples 3

B. Experimental procedure 3

C. Quantification of hyperpolarization 4

D. MRI control code 4

1. Example of PH-INEPT+ pulse program for Paravision 6 4

2. Valve control script VentilSTRG.incl 6

Supplementary Note 1 10

Supplementary Note 2 11

References 15

Supplementary Methods

A. Preparation of samples

Parahydrogen was enriched to ≈ 85 % (Supplementary Figure 1), stored in aluminum cylinders and was used on demand as described elsewhere [1]. Note that this converter produced ~99% parahydrogen previously. The lower enrichment may be caused by contaminations of the catalyst chamber, the cylinders or the connecting tubes but has not been investigated yet.

Reaction solutions were prepared by mixing 5 mM substrate precursor and 2 mM catalyst in deionized water using the following substances: **Precursors** – hydroxyethyl acrylate-1-^{13}C,2,3,3-d_3 (HEA, 98 atom % D, 99 atom % ^{13}C, *MW* = 120.13 g·mol^{-1},CAS: 1216933-17-3, Sigma Aldrich, USA) ; or fumaric acid-1-^{13}C,2,3-d_2 (FUM, 98 atom % D, 99 atom % ^{13}C, *MW* = 119.08 g·mol^{-1}, CAS: 1018681-16-7, Cambridge Isotope Laboratories, USA). **Rhodium-based water-soluble catalyst** – formed by mixing a bisphosphine ligand (1,4-bis-[(phenyl-3-propane sulfonate) phosphine] butane disodium salt, *MW* = 562.53 g·mol^{-1}, MDL number: MFCD15144866, Q36333, Sigma Aldrich, USA) and a rhodium moiety (bis(norbornadiene)-rhodium (I) tetrafluoroborate, *MW* = 373.99 g·mol^{-1}, CAS: 36620-11-8, StremChemicals, MA,USA); the ligand was added in 10 % excess compared to the rhodium complex, i.e., at a concentration of 2.2 mM. In fumaric acid samples, a **phosphate buffer** was used to adjust the pH value of the final catalyst substrate solution to 2.9 (40 mM potassium dihydrogen phosphate, KH_2PO_4, CAS: 7778-77-0; and ≈10 mM phosphoric acid, H3PO4, CAS: 7664-38-2).[2]

During the experiment, HEA was reacted to hydroxyethyl propionate-1-^{13}C,2,3,3-d_3 (HEP), and FUM to succinic acid-1-^{13}C,2,3-d_2 (SUC).

B. Experimental procedure and valve actuation

In the following, valves V1-5 and Syringes S1-2 refer to the devices schematically shown in Supplementary Figure 3. The experiment consisted of the following steps:

1. The reaction chamber was flushed (and emptied) with N_2 gas (5-7 bar) by opening the corresponding valves V3, V4, and V5, as well as syringe S1 (which was opened manually) in Supplementary Figure 3).
2. S1 was closed and the gas outlet of the reactor (V2) was opened. 1 mL of catalyst-substrate solution was injected manually into the reactor within 5 s.
3. pH_2 was injected into the reaction solution from the bottom of the reactor for 2 s (V1 open).
4. Hydrogenation took place for another 3 s (V3 open). When the Goldman sequence was used (see next step), ^{1}H decoupling was played out during this time (MLEV-16 scheme; power level was 50 W).[3]

5. Spin order transfer (SOT) was performed with PH-INEPT+ sequence[4] for HEP, or Goldman's sequence[5] when SUC was polarized.
6. Hyperpolarized ^{13}C signal was detected at the end of the SOT (2048 real data points, spectral width of 2562 Hz / 34 ppm, receiver gain of 203, acquisition time of 799.54 ms).

This procedure was repeated every 15-s.

C. Quantification of hyperpolarization

In vitro quantification was achieved by comparing the signals of the hyperpolarized sample with that of a reference solution containing naturally abundant ^{13}C at thermal equilibrium (5 ml acetone, ^{13}C fraction of ~1.1%). It was quantified with respect to the concentration of added precursor (not only the hydrogenated fraction) and assuming 700µL solution was left in the reactor. As the reactor was filled manually using a 10 ml syringe the error of inserted volume was estimated to $\Delta V = \pm 100$ µl, but this deviation was not considered for the quantification. Moreover, the incomplete enrichment of pH_2 (section A) and a potentially incomplete hydrogenation were not considered in the quantification. In other words, reported polarizations could be further improved if 100 % pH_2 were used and if the precursor were fully hydrogenated within the same time (5 s).

D. MRI control code

1. Example of PH-INEPT+ pulse program for Paravision 6

```
;****************************************************************
; Edited by M. Zimmermann and A. Schmidt
;
; based on
;
; SINGLEPULSE - non-selective spectroscopy (pulse + acquire)
;
; Copyright (c) 2002 - 2003
; Bruker BioSpin MRI GmbH
; D-76275 Ettlingen, Germany
;
; All Rights Reserved
;
;****************************************************************
;
; d0 - TR padding
; d8 - SCON / BLKTR_MAN (Amplifier preblanking)

#include <MRI.include>
#include <TriggerDef.mod>
#include <acqdec.mod>
#include <NoeDef.mod>
#include <VentilSTRG.incl>
define delay dur1
"dur1=d1-de"
define loopcounter lds={$DS}
preset off
```

```
; SOT timings
define delay evoInt1={$EvoInt1}
"evoInt1_half=evoInt1*0.5"
define delay evoInt2={$EvoInt2}
"evoInt2_half=evoInt2*0.5"
; valve control timings
define delay BubblingTime={$BubblingTime}
define delay BypassTime={$BypassTime}


INIT_DEVICES
5u    rpp0
;----------------------------------start of the main loop ----------
start,      20u     reload B0

;----------------------------------preparation modules -------------
d0

V3_V4_on ; equilibrate pressure at 1bar in the entire system
1s

5u trignl1 ; manual trigger for when solution is ready and reactor placed

; Empty reactor
V3_V4_V5_on
4s
; Fill reactor
V2_on
5s
; pH2 filling
V1_on
2s
; Equilibrate reactor inlet and outlet
V3_on
3s

; start SOT sequence

;f1 = 13C , f2 = 1H
;ph0 = phase x, ph1 = phase y

;Pulse 45y 1H
d8 gatepulse 2
(p0:sp0 ph1):f2

;evolution delay t1/2
evoInt1_half

;Echo Pulses 180 1H+13C
d8 gatepulse 1|2
(center (p6:sp6):f1 (p2:sp2):f2)

;evolution delay t1/2
evoInt1_half

;Pulses 90y 1H and 90x 13C
d8 gatepulse 1|2
(center (p5:sp5 ph0):f1 (p1:sp1 ph1):f2)

;evolution delay t2/2
evoInt2_half

;Echo Pulses 180 1H+13C
(center (p6:sp6):f1 (p2:sp2):f2)
```

```
;evolution delay t2/2
evoInt2_half


subr acqdec_jobs(ph30,ph31)

; ADC_INIT(ph0, ph1)
; aqq ADC_START

5m
10u     ADC_END
V_off
;
"lds = lds - 1"         ; this makes
if "lds>=0" goto start  ; dummy scans
1m    ipp0


lo to start times NA
        2.5u      rpp0
lo to start times NR
SETUP_GOTO(start)

exit

ph0 = 0
ph1 = 1
ph2 = 2
ph3 = 3
ph31 = 0 ; receiver
ph30 = 0 ; reference
```

2. Valve control script VentilSTRG.incl

```
;*******************************************************************
; Written by M. Zimmermann, 2018
;
; UNIVERSITY MEDICAL CENTER FREIBURG
; Department of Radiology - Medical Physics
; Killianstr. 5a · 79106 Freiburg;
;
; this file includes the valve commands
; If a new combination is required create it via #define and
; blank/unblank [30] on the desired positions. Do not change [29]
; and [31] {[29] enables/disables sweeping and [31] ist
; the sweeping/clock itself}
; DO NOT use ANY number but [28, 29, 30 ,31]=TTL_output[1, 2, 3, 4].
; The others are used/reserved by commands controling the gradients etc.
;
;*******************************************************************
define delay tog
"tog=15u" ;set the time needed to blank or unblank the TTL outputs
; enable sweep load V1
;--------------------------------------------------------------------------------

#define V_off tog setnmr3|29 \n tog
#define V2_V5_on tog setnmr3|29 \n tog
check V1 reset load V2
---------------------------------------------------------------------------------
\n tog setnmr3|31 \n tog setnmr3^31 \n tog
\n tog setnmr3|31 \n tog setnmr3^31 \n tog setnmr3|30
check V2 reset load V3
---------------------------------------------------------------------------------
\n tog setnmr3|31 \n tog setnmr3^31 \n tog
\n tog setnmr3|31 \n tog setnmr3^30^31 \n tog
check V3 reset load V4
```

```
-----------------------------------------------------------------------
\n tog setnmr3|31 \n tog setnmr3^31 \n tog
\n tog setnmr3|31 \n tog setnmr3^31 \n tog
check V4 reset load V5
-----------------------------------------------------------------------
\n tog setnmr3|31 \n tog setnmr3^31 \n tog
\n tog setnmr3|31 \n tog setnmr3^31 \n tog setnmr3|30
check V5 reset load V6
-----------------------------------------------------------------------
\n tog setnmr3|31 \n tog setnmr3^31 \n tog
\n tog setnmr3|31 \n tog setnmr3^30^31 \n tog
check V6 reset load V7
-----------------------------------------------------------------------
\n tog setnmr3|31 \n tog setnmr3^31 \n tog
\n tog setnmr3|31 \n tog setnmr3^31 \n tog
check V7 reset load V8
----------------------------------------------------------------
\n tog setnmr3|31 \n tog setnmr3^31 \n tog
\n tog setnmr3|31 \n tog setnmr3^31 \n tog
check V8 reset and stop sweep
----------------------------------------
\n tog setnmr3|31 \n tog setnmr3^29^31
\n tog setnmr3|31 \n tog setnmr3^29^31
```

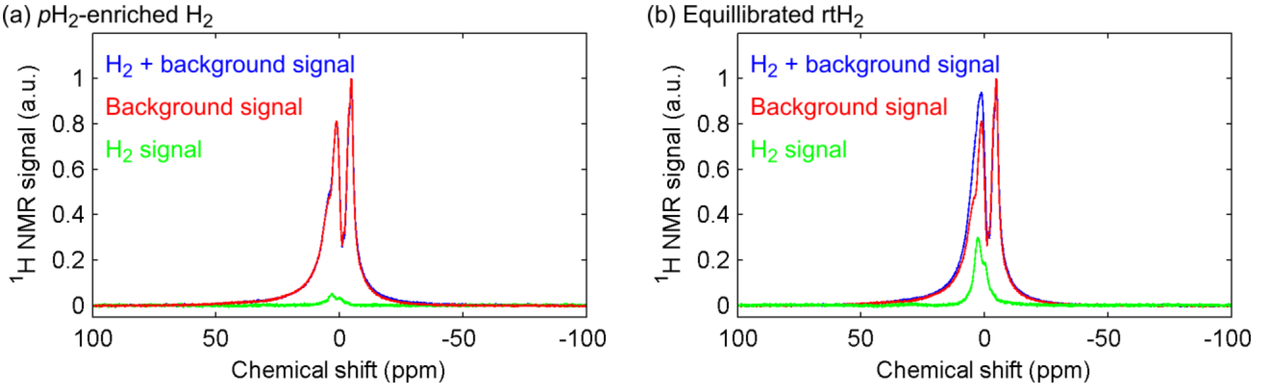

Supplementary Figure 1: Enrichment of parahydrogen. Gaseous N_2, *p*H_2-enriched H_2 and room-temperature H_2 (rtH_2) were filled into a glass vial at 1 bar and non-localized ^{1}H-NMR was acquired (α = 9°, N = 256, acquisition time of 128 ms, T_R = 200 ms, B_0 = 7 T). N_2 and *p*H_2 yield no signal, thus, the measured signals correspond to a composition of the *ortho*hydrogen fraction (green) and background signals (red). The H_2 signals of the *p*H_2 enriched sample (a, blue) and the rtH_2 sample (b, blue) were isolated by subtracting the background signal and comparing the residuals,[1] which suggested the enriched sample to have a *p*H_2 fraction of ≈85 % compared to 25 % without enrichment at room temperature.

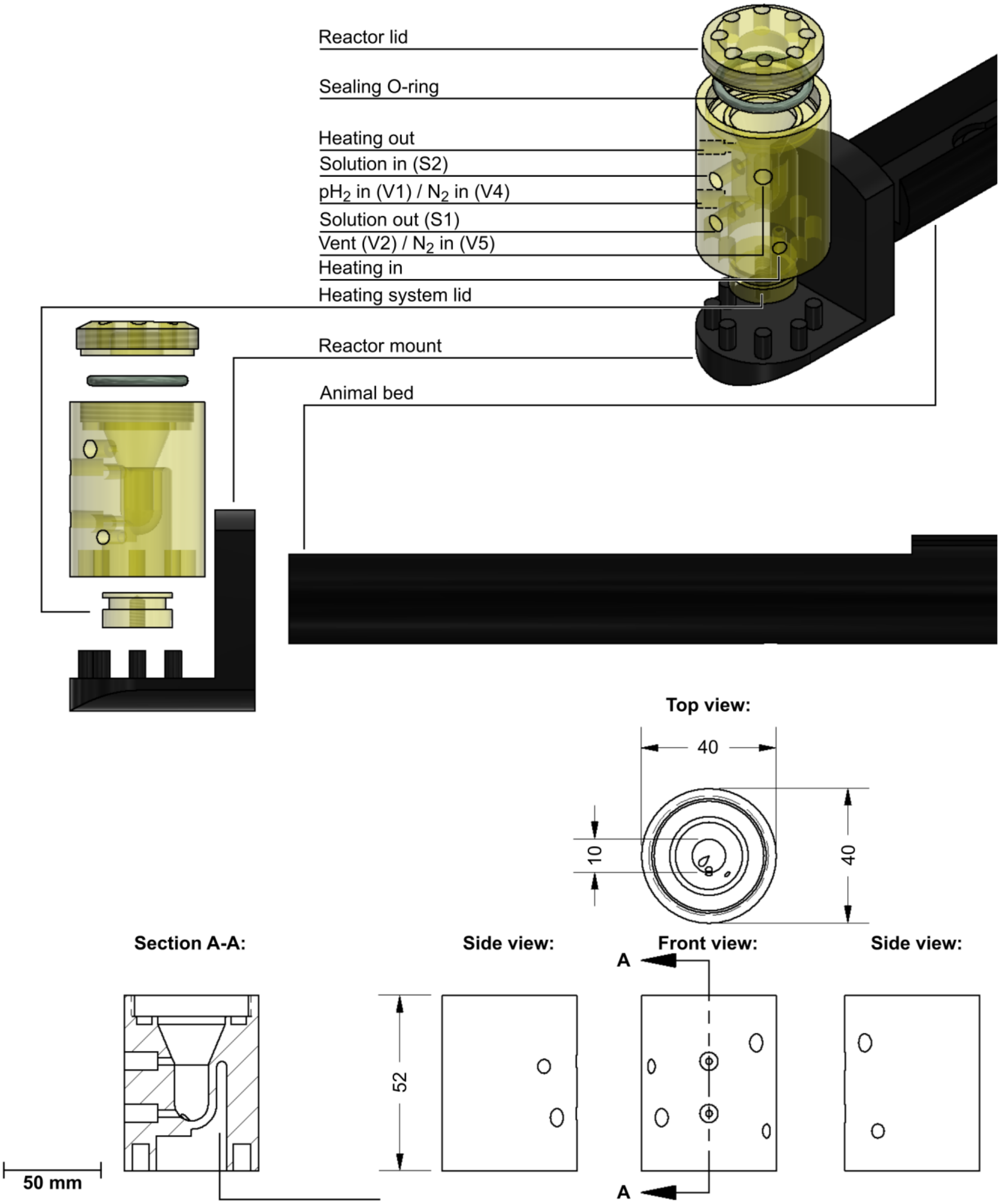

Supplementary Figure 2: Technical drawing of the reactor. The reactor, which was constructed out of polysulfone, is depicted together with the mount and animal bed (top). The projections from the top, sides and front and a vertical cross section (Section A-A) show the connections, heating- and inner reaction chamber (bottom). V1 – V5 and S1 and S2 refer to valves and syringes as indicated in Supplementary Figure 3. pH_2 and N_2 refer to parahydrogen-enriched gas and nitrogen gas, respectively.

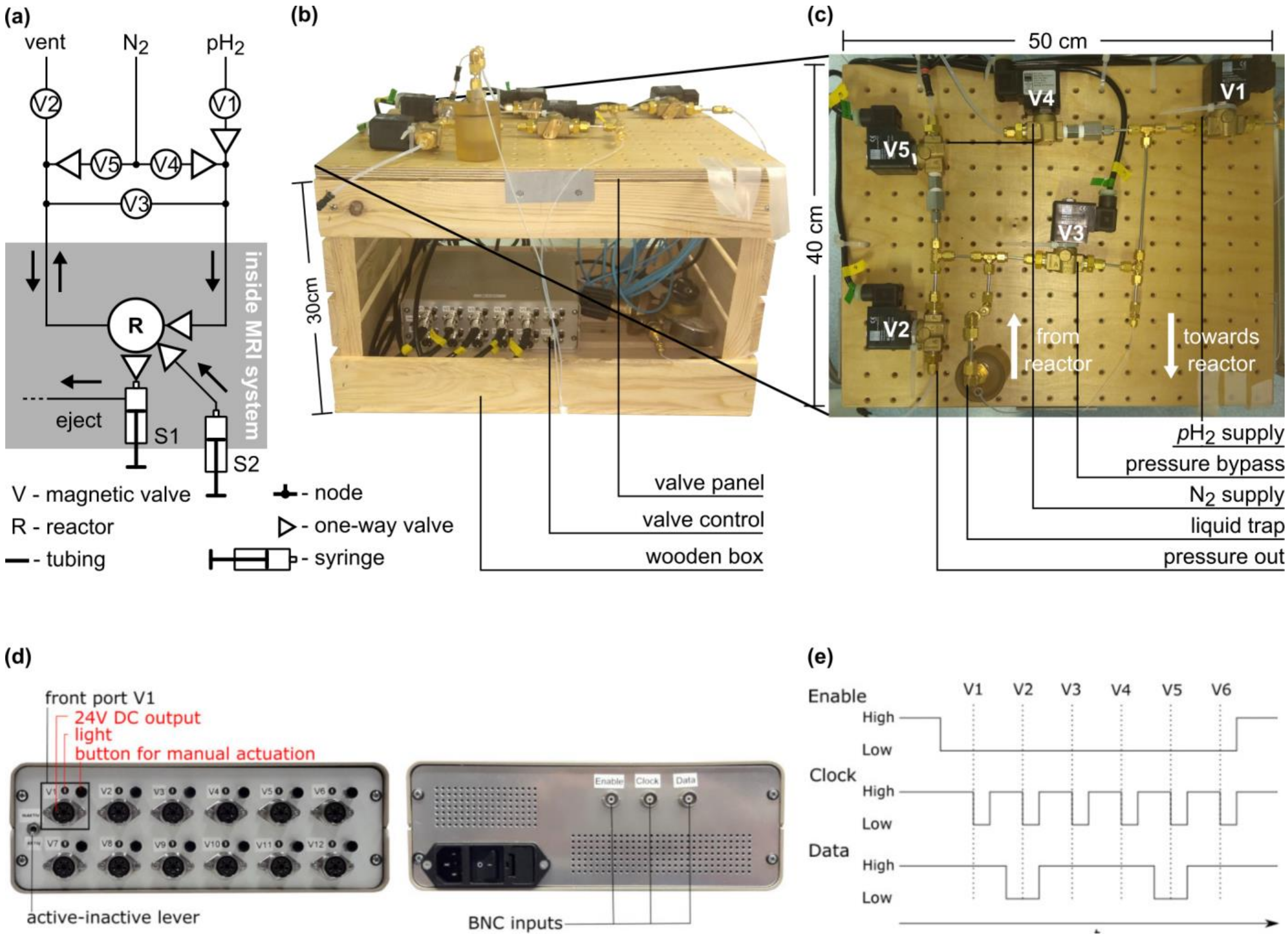

Supplementary Figure 3: Fluid control. (a) The HP experiment and flow of the pH_2, N_2 and solution were controlled by magnetic valves (V1 – V5), one-way valves (triangles) and syringes (S1 and S2). The valves and the reactor (R) were connected with 1/16” ETFE or 1/8” stainless steel tubes. V1 was opened to deliver pH_2 to the reactor. V2 was used to release pressure. V3 allowed equilibrating the pressure between the gas in- and output of the reactor and stopped bubbling. V4 and V5 enabled flushing the system with N_2 gas and removed the solution from the reactor. The syringes were used to inject a fresh catalyst-precursor solution into the reactor (S2) or to eject a hyperpolarized agent (S1). (b) Photographs of the fluid control (b) and a top view of the valve panel (c) are shown; the entire transportable setup (without the MRI system) fitted into a 50 x 40 cm^2 box and had a total weight of ≈5 kg. (d) The magnetic valves were operated from the pulse program of the MRI system using its digital outputs and a Teensy 3.5 microcontroller that controlled a relay box. (e) A signal series generated at the digital outputs of the MRI scanner actuated the valves as required, which is exemplarily shown for an activation of valves V2 and V5 while V1, V3, V4 and V6 were kept close: The three binary outputs (Enable, Clock and Data) are active LOW (0 Volt) and, therefore, were kept HIGH (5 Volts) by default. One actuating series started when “Enable” was switched to LOW. Each “switch” of “Clock” corresponds to a front port (V1-V6; note only five valves were needed here). A valve was switched on when “Data” was LOW and off when “Data” was HIGH during the corresponding “Clock” signal.

Supplementary Note 1

Lifetime of hyperpolarization: The hyperpolarization decays with the longitudinal relaxation time, T_1. Supplementary Figure 5 reports the ^{13}C T_1 of hyperpolarized [1-13C]HEP-d3 in water and D_2O.

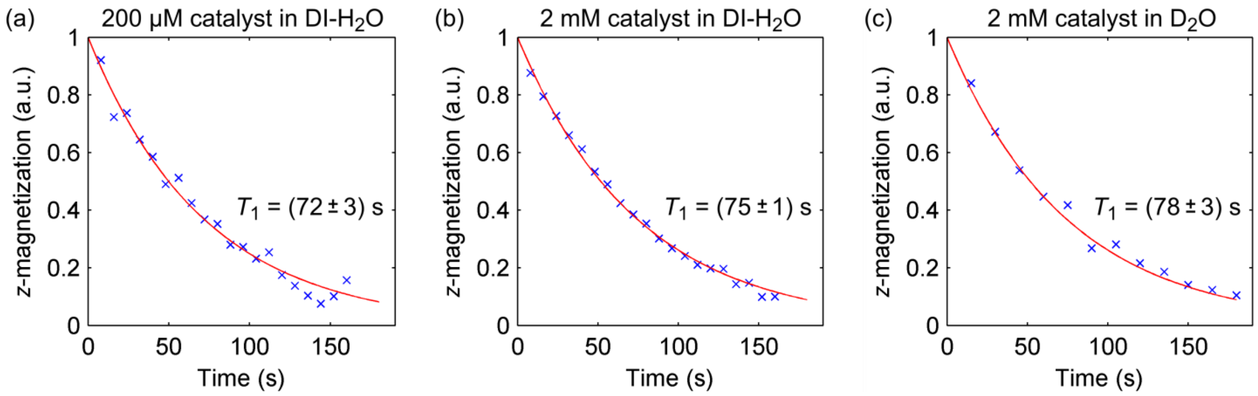

Supplementary Figure 4 | Longitudinal relaxation of hyperpolarized HEP-1-^{13}C. The decaying longitudinal magnetization was measured using ^{13}C NMR with low flip-angle excitations at 7 T and 80 °C in different aqueous solutions: (a) with a catalyst concentration of c_{cat} = 200 µM in deionized H_2O, (b) with c_{cat} = 2 mM in deionized H_2O and (c) with c_{cat} = 2 mM in D_2O. The measured data is represented by blue crosses. T_1 values were obtained by fitting an exponential decay function to the data (red solid line, all $R^2 > 0.97$). (a) and (b): repetition time T_R = 8 s, flip angle α = 10 °, c_{HEA} = 10 mM, hydrogenation time t_h = 5 s. (c): T_R = 15 s, α = 9 °, c_{cat} = 2 mM, c_{HEA} = 5.54 mM in D_2O, t_h = 5 s. Data is reported in Supplementary Table 4. Data in (c) has been published before.[6]

Supplementary Note 2

Adjusting temperature of the produced samples: The temperature of repetitively ejected hot solution was measured. Ten 500 µL samples of 80 °C hot H_2O were drawn up with a syringe and ejected subsequently through a 20 cm catheter (fine bore polyethylene tubing, 0.61 mm OD x 0.28 mm ID, Smiths Medical International Ltd., UK) isochronously at a temporal distance of 15 s. Due to heat exchange with the catheter, syringe, associated fittings, and ambient air, the temperature was found to drop to 33 °C in the first ejection but then increased over the repetitions up to 48 °C in the last one. The mean with standard deviation of the temperature was (39 ± 14) °C (Supplementary Table 1). The experiment was repeated, but this time the injection catheter was passively cooled by guiding ≈5 cm of it through cold water (≈16 °C). Here, the temperature of the ten injections was almost constant at (32 ± 2) °C (Supplementary Table 1).

Supplementary Table 1 | Measured temperatures of the solution ejected from a hot reservoir (80 °C).

Injection Nº	Time / s	Temperature of injection / °C	
		Cooling 1 (air)	Cooling 2 (16 °C water)
1	15	33	29
2	30	41	32
3	45	43	29
4	60	41	30
5	75	40	32
6	90	45	31
7	105	46	33
8	120	43	33
9	135	47	34
10	150	48	32
	Mean :	39	32
	Standard deviation :	14	2

Supplementary Table 2 | Data reported in Figure 1. Exp No: experiment number; P13C: ^{13}C polarization; std: standard deviation; st error: standard error of the mean.

(a) HEP

Exp No	time / s	P13C / %
1	15	15,7
2	30	14,4
3	45	25,0
4	60	26,6
5	75	18,7
6	90	20,9
7	105	20,7
8	120	20,1
9	135	15,2
10	150	15,3
	mean / %	19,3
	std / %	4,0
	st error / %	1,3

(b) SUC

Exp No	time / s	P13C / %
1	15	1,2
2	30	1,8
3	45	1,8
4	60	2,5
5	75	2,0
6	90	2,4
7	105	1,0
8	120	1,3
9	135	1,2
	mean / %	1,7
	std / %	0,5
	st error / %	0,2

Supplementary Table 3 | Data reported in Figure 3. T: temperature; p: pressure; ccat: concentration of the catalyst; cHEA: concentration of the substrate precursor; th: hydrogenation time; P13C: ^{13}C polarization; std: standard deviation; st error: standard error of the mean.

(a) P13C(T)

T / °C	P13C / %	mean / %	std / %	st error / %
22,0	4,8			
22,0	6,1			
22,0	5,0	5,3	0,7	0,4
40,0	8,7			
40,0	8,9			
40,0	7,3	8,3	0,8	0,5
60,0	11,3			
60,0	12,1			
60,0	10,9	11,4	0,6	0,3
80,0	14,7			
80,0	14,8			
80,0	14,8	14,8	0,1	0,0
90,0	17,7			
90,0	15,9			
90,0	15,1	16,2	1,4	0,8
97,2	14,3			
97,2	16,9			
97,2	16,8	16,0	1,5	0,8

(b) P13C(p)

p / bar	P13C / %	mean / %	std / %	st error / %
5,0	2,0			
5,0	2,7			
5,0	3,6	2,8	0,8	0,4
7,5	6,9			
7,5	6,9			
7,5	6,6	6,8	0,2	0,1
10,0	10,7			
10,0	11,0			
10,0	10,7	10,8	0,2	0,1
12,5	11,8			
12,5	12,3			
12,5	11,9	12,0	0,3	0,2

15,0	12,4			
15,0	14,8			
15,0	14,3	13,8	1,2	0,7
17,5	16,2			
17,5	15,6			
17,5	16,3	16,0	0,4	0,2
20,0	16,3			
20,0	16,0			
20,0	17,4	16,5	0,7	0,4

(c) P13C(ccat)

ccat / mM	P13C / %	mean / %	std / %	st error / %
1,0	4,98			
1,0	4,07			
1,0	4,4	4,5	0,5	0,3
2,0	3,2			
2,0	4,83			
2,0	4,54	4,2	0,9	0,5
3,0	2,99			
3,0	3,17			
3,0	5,91	4,0	1,6	0,9
4,0	5,15			
4,0	4,25			
4,0	4,34	4,6	0,5	0,3

(d) P13C(cHEA)

cHEA / mM	P13C / %	mean / %	std / %	st error / %	payload / 1	std / 1	st error / 1
5,0	14,7						
5,0	16,3						
5,0	18,1	16,4	1,7	1,0	0,17	0,02	0,01
10,0	19,7						
10,0	16,8						
10,0	18,1	18,2	1,4	0,8	0,37	0,03	0,02
20,0	10,6						
20,0	9,8						
20,0	13,0	11,1	1,7	1,0	0,45	0,07	0,04
40,0	9,4						
40,0	10,2						
40,0	9,0	9,5	0,6	0,3	0,78	0,06	0,04
80,0	6,3						
80,0	5,3						
80,0	5,9	5,8	0,5	0,3	0,95	0,09	0,05

(e)

th / s	P13C / %	mean / %	std / %	st error / %
2	12,08			
2	12,81			
2	14,27	13,1	1,1	0,6
3	15,71			
3	13,86			
3	15,64	15,1	1,0	0,6
4	18,09			
4	22,19			
4	21,89	20,7	2,3	1,3
5	23,85			
5	25,12			
5	24,96	24,6	0,7	0,4

6	23,26			
6	20,93			
6	18,65	20,9	2,3	1,3
7	20,10			
7	21,06			
7	16,30	19,2	2,5	1,5
8	17,33			
8	14,98			
8	20,78	17,7	2,9	1,7
9	18,26			
9	16,93			
9	15,06	16,8	1,6	0,9
11	17,63			
11	14,84			
11	14,40	15,6	1,8	1,0
13	13,77			
13	13,82			
13	16,84	14,8	1,8	1,0
15	12,10			
15	12,80			
15	14,28	13,1	1,1	0,6

Supplementary Table 4 | Data reported in Supplementary Figure 4. P13C: ^{13}C polarization normalized to P13C(t=0s) as predicted from an exponential fit.

(a)

time / s	P13C / norm to t=0
8	0,92
16	0,72
24	0,74
32	0,65
40	0,59
48	0,49
56	0,51
64	0,42
72	0,37
80	0,35
88	0,28
96	0,27
104	0,23
112	0,25
120	0,17
128	0,14
136	0,10
144	0,08
152	0,10
160	0,16

(b)

time / s	P13C / norm to t=0
8	0,88
16	0,79
24	0,73
32	0,66
40	0,61
48	0,53
56	0,49
64	0,42
72	0,39
80	0,35
88	0,30
96	0,27
104	0,24
112	0,21
120	0,20
128	0,20
136	0,14
144	0,15
152	0,10
160	0,10

(c)

time / s	P13C / norm to t=0
15	0,84
30	0,67
45	0,54
60	0,45
75	0,42
90	0,27
105	0,28
120	0,22
135	0,19
150	0,14
165	0,12
180	0,10

References

1. Hoevener J-B, Bär S, Leupold J, Jenne K, Leibfritz D, Hennig J, et al. A continuous-flow, high-throughput, high-pressure parahydrogen converter for hyperpolarization in a clinical setting. NMR in Biomedicine. 2013;26: 124–131.

2. Chekmenev EY, Hövener J, Norton VA, Harris K, Batchelder LS, Bhattacharya P, et al. PASADENA Hyperpolarization of Succinic Acid for MRI and NMR Spectroscopy. J Am Chem Soc. 2008;130: 4212–4213. doi:10.1021/ja7101218

3. Berner S, Schmidt AB, Zimmermann M, Pravdivtsev AN, Glöggler S, Hennig J, et al. SAMBADENA Hyperpolarization of 13C-Succinate in an MRI: Singlet-Triplet Mixing Causes Polarization Loss. ChemistryOpen. 2019;8: 728–736. doi:10.1002/open.201900139

4. Haake M, Natterer J, Bargon J. Efficient NMR Pulse Sequences to Transfer the Parahydrogen-Induced Polarization to Hetero Nuclei. J Am Chem Soc. 1996;118: 88–91.

5. Goldman M, Johannesson H. Conversion of a proton pair para order into 13C polarization by rf irradiation, for use in MRI. Comptes Rendus Physique. 2005;6: 575–581.

6. Schmidt AB, Berner S, Schimpf W, Müller C, Lickert T, Schwaderlapp N, et al. Liquid-state carbon-13 hyperpolarization generated in an MRI system for fast imaging. Nature Communications. 2017;8: 14535. doi:10.1038/ncomms14535
